# Opioid-Free Anesthesia Benefit–Risk Balance: A Systematic Review and Meta-Analysis of Randomized Controlled Trials

**DOI:** 10.3390/jcm10102069

**Published:** 2021-05-12

**Authors:** Arthur Salomé, Hakim Harkouk, Dominique Fletcher, Valeria Martinez

**Affiliations:** 1 Service d’anesthésie, Hôpital Ambroise Paré et Raymond Poincaré, Boulogne Billancourt et Garches, Assistance Publique Hôpitaux de, 92380 Paris, France; arthur-salome@orange.fr (A.S.); hakim.harkouk@aphp.fr (H.H.); dominique.fletcher@aphp.fr (D.F.); 2 Department of Anesthesia, Université Paris-Saclay, UVSQ, Inserm, LPPD, 92100 Boulogne, France

**Keywords:** opioid-free anesthesia, adverse effect, anesthesia, analgesia, systematic review

## Abstract

Opioid-free anesthesia (OFA) is used in surgery to avoid opioid-related side effects. However, uncertainty exists in the balance between OFA benefits and risks. We searched for randomized controlled trials (RCTs) comparing OFA to opioid-based anesthesia (OBA) in five international databases. The co-primary outcomes were postoperative acute pain and morphine consumption at 2, 24, and 48 h. The secondary outcomes were the incidence of postoperative chronic pain, hemodynamic tolerance, severe adverse effects, opioid-related adverse effects, and specific adverse effects related to substitution drugs. Overall, 33 RCTs including 2209 participants were assessed. At 2 h, the OFA groups had lower pain scores at rest MD (0.75 (−1.18; −0.32)), which did not definitively reach MCID. Less morphine was required in the OFA groups at 2 and 24 h, but with very small reductions: 1.61 mg (−2.69; −0.53) and −1.73 mg (*p* < 0.05), respectively, both not reaching MCID. The reduction in PONV in the OFA group in the PACU presented an RR of 0.46 (0.38, 0.56) and an RR of 0.34 (0.21; 0.56), respectively. Less sedation and shivering were observed in the OFA groups with an SMD of −0.81 (−1.05; −0.58) and an RR of 0.48 (0.33; 0.70), respectively. Quantitative analysis did not reveal differences between the hemodynamic outcomes, although severe side effects have been identified in the literature. No clinically significant benefits were observed with OFA in terms of pain and opioid use after surgery. A clear benefit of OFA use was observed with respect to a reduction in PONV. However, more data on the safe use of OFAs should be collected and caution should be taken in the development of OFA.

## 1. Introduction

Opioids have long been used to supplement general anesthesia. However, opioids are associated with well-recognized side effects such as nausea and vomiting, sedation, ileus, confusion and delirium, respiratory depression, increased postoperative pain and morphine consumption, immunodepression, hyperalgesia and chronic postoperative pain, addiction, and misuse [1]. The recent opioid crisis, especially in the United States, has further questioned the perioperative use of opioids [2]. The common practice of administering opioids during anesthesia was therefore challenged by clinical studies suggesting that opioid-free anesthesia (OFA) may be effective in providing adequate pain control, while reducing postoperative opioid consumption and hopefully reducing opioid-related side effects [3]. Intuitively, opioids should thus be avoided during surgery and should be replaced by hypnotic or analgesic drugs to control the consequences of surgical trauma during anesthesia, but this suggestion has never been validated. Although the definition of OFA varies in the literature and between centers, lidocaine, ketamine, and alpha-2 agonists such as clonidine or dexmedetomidine have been proposed to replace opioids alone or in combination [3].

Previous studies on this subject have been heterogeneous with small sample sizes, suggesting fragile and inconsistent benefits. Recent meta-analyses have suggested limited benefits pertaining to the incidence of postoperative pain, nausea, and vomiting [4,5,6]. The weaknesses and methodological limitations of these meta-analyses have been highlighted [3,7]. Therefore, the lack of high-level evidence leaves clinicians uncertain about whether OFA is beneficial or harmful. Alpha-2-receptor agonists frequently used for this purpose, such as dexmedetomidine, may promote unwanted side effects, including increased risks of hypotension and bradycardia during surgery and prolonged sedation after surgery [8]. In addition, in the recent, largest randomized trial analyzing the outcomes after OFA, several cases of severe bradycardia were described in the OFA group, suggesting that clinicians should be cautious about the safety of OFA [9].

To address this uncertainty, we conducted a systematic review and meta-analysis to evaluate the benefits and risks related to OFA compared to opioid-based anesthesia. Our analysis is based on a clinical significance perspective to evaluate the level of confidence in the benefit–risk ratio of OFA in existing data.

## 2. Methods

### 2.1. Data Sources and Search Strategy

This systematic review of randomized controlled trials (RCTs) and observational studies was performed in accordance with the criteria of the PRISMA statement and the current recommendations of the Cochrane Collaboration [10]. We searched the CENTRAL, MEDLINE, and EMBASE databases for relevant studies from the inception of the database until April 2021, with no limitations on publication language or status, using the terms “opioid-free”, “opiate-free”, “anesthesia” or “anesthetic”, and “surgery” or “surgical”. We identified RCTs using the highly sensitive search strategy of the Cochrane Collaboration. We also searched the Database of Abstracts of Reviews of Effects for previous, relevant systematic reviews and randomized included trials, and ClinicalTrials.gov and the WHO International Clinical Trials Registry Platform for completed trials.

### 2.2. Eligibility Criteria

Randomized controlled trials comparing “opioid-free anesthesia” (OFA group) to “opioid-based anesthesia” (OBA group) were included. Trials performed in adults (defined as 18 years of age and older for at least 80% of the study population) undergoing elective or emergent surgery under general anesthesia were considered. The trials included must have evaluated “opioid-free anesthesia”, regardless of the drugs used, the doses, or the combination of drugs. The unique condition was the total absence of opioid use during general anesthesia; a single dose of opioid during induction was acceptable. A standardized general anesthesia in the two groups was a prerequisite. We included RCTs that evaluated the postoperative benefit. At least one outcome of interest had to be assessed to be considered for inclusion. We included articles published in English, French, Spanish, or Dutch. Trials were excluded when regional analgesia (neuraxial, peripheral, and infiltration) was present and when participants concurrently took opioids for another condition.

Two authors (A.S. and D.F.) independently screened the titles, abstracts, and complete manuscripts according to the selection criteria. Any disagreement was discussed with a third author (V.M.) until a consensus was reached.

### 2.3. Data Extraction and Assessment of the Risk of Bias

Two reviewers (A.S. and V.M.) independently extracted data from each study. Disagreements were resolved by consensus with a third reviewer (D.F.). Information about the trials (first author, year of publication, country, number of groups in the study, and sponsorship), participants (characteristics of the population, and number of patients randomized and analyzed), and experimental intervention (drug used and doses) was extracted. Two independent reviewers (A.S. and D.F.) assessed the quality of the trial methodology with the Cochrane Risk of Bias tool, and any discrepancies were resolved by consensus.

### 2.4. Outcome Measures

The co-primary outcomes were postoperative acute pain and morphine consumption at 2, 24, and 48 h. The secondary outcomes were the incidence of postoperative chronic pain (defined as pain lasting for 3 months or more), hemodynamic tolerance assessed by mean heart rate and mean arterial pressure at 30 min after induction and at the arrival in the recovery room, incidence of severe adverse effects (SAE) within the first 24 h, adverse effects related to opioid use (nausea, vomiting, and sedation), and specific adverse effects related to a substitution drug. Most studies did not distinguish between nausea and vomiting [11] and reported both outcomes together. We therefore used the classification defined in the article by Apfel and colleagues [12] to determine the incidence of nausea. We considered hemodynamic instability as an SAE when drug administration was required (atropine for bradycardia, vasoactive drug for hypotension or hypertension, and beta blockers for tachycardia). We considered respiratory depression as defined by those authors (decrease in respiratory rate or hypoxemia).

### 2.5. Data Synthesis and Analysis

Dichotomous outcomes were extracted as the presence or absence of an effect. For continuous data, we extracted the means and standard deviations (SD). If not reported, the standard deviations were obtained from the confidence intervals or *p* values for the differences between the means of two groups [13,14]. If medians with ranges were reported, we obtained the mean and standard deviation using the method described by Hozo [15]. If only means were reported, we contacted the authors. If the authors did not respond, we took the respective median standard deviations of each group. The authors of the original study were contacted (by V.M.) and asked to provide missing data or to add data when possible. If necessary, the means and measures of dispersion were approximated from figures generated with a dedicated software called webplotdigitizer. For studies in which more than two groups with different doses of intraoperative opioids were compared, we combined the results following the recommendation of the Cochrane handbook. The pain intensity measurement scores were collected using a scale from 0 (no pain) to 100 (worst imaginable pain) points. When scores were not presented in a 100-point scale format, they were converted (mean and SD) using the appropriate ratio. All pain scores reported within 1 h of the time points of interest, as specified above, were included in the analysis. Pain intensity scores were assumed to be at rest unless otherwise noted. The minimal clinically important difference (MCID) between groups for acute pain intensity was established as 10 points on a 100-point scale and is independent of pain severity [16]. A difference of 20 to 30 points represents an “appreciable” analgesic effect, while a 50-point difference represents a “substantial” effect [16]. For comparison of opioid administration, all doses of opioids were converted to intravenous milligrams of morphine equivalents using the data from a recent recommendation [17].

The minimal clinically important difference between groups for morphine consumption was established at 10 mg at 24 h based on a review of the literature [18]. We calculated the risk ratios (RR) with 95% confidence intervals (CI) for the dichotomous data and the mean differences (MD) with 95% CI for the continuous data. We expected heterogeneity among the data (because of the diverse populations included); therefore, we used the Dersimonian and Lairs random effects meta-analysis modules. We assessed heterogeneity with the I^2^ statistic (I^2^ > 50% indicates substantial heterogeneity). Subgroup and sensitivity analyses were defined a priori to evaluate potential sources of heterogeneity. The subgroup analyses were the type of OFA (opioid substitution or nothing), the hypnotic used (propofol vs. halogen), the use of a patient-controlled analgesia pump, and the use of opioids for induction.

### 2.6. Strength of Evidence

We present the primary outcomes of this review in the “Summary of findings” tables, as recommended in the Cochrane Handbook for Systematic Reviews of Interventions. The quality of evidence for each outcome was rated according to the Grades of Recommendation, Assessment, Development, and Evaluation (GRADE) Working Group system [19] for five criteria: risk of bias, inconsistency, indirectness, imprecision, and publication bias. Each point was rated independently by two authors (V.M. and A.S.), with discussion to reach a consensus if necessary. 

## 3. Result

### 3.1. Search Results

**Figure 1:**
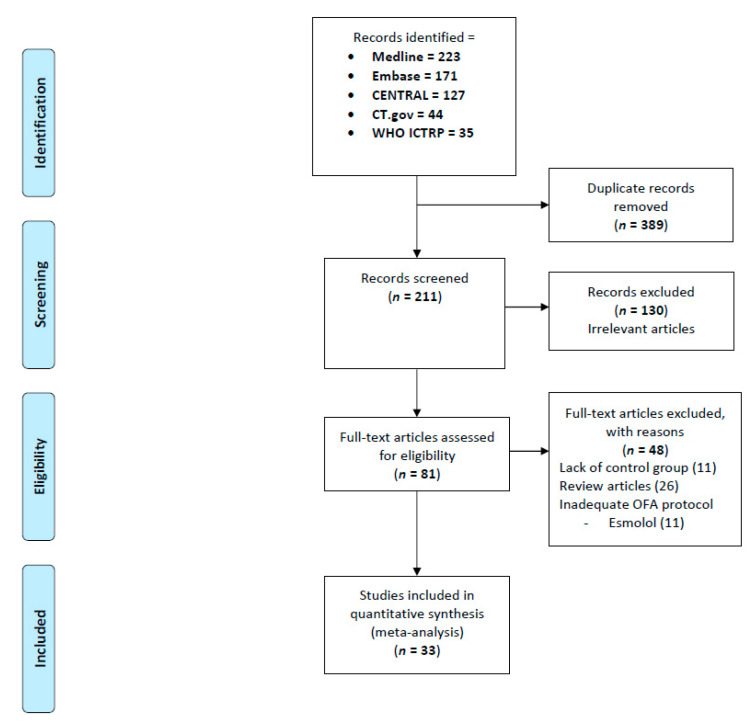
PRISMA flow chart detailing retrieved, excluded, assessed, and included trials.

The systematic literature search identified 600 relevant publications. After a review of the titles and abstracts, 81 studies were selected and assessed for potential inclusion into this systematic review. After reading the full-text articles, 33 RCTs (published between 1994 and 2021) including 2209 participants were finally included (Figure 1) [20,21,22,23,24,25,26,27,28,29,30,31,32,33,34,35,36,37,38,39,40,41,42,43,44,45,46,47,48,49,50,51,52]. No unpublished trials were identified with our eligibility criteria in the clinicaltrial.gov register.

### 3.2. Trial, Participants, and Intervention Characteristics

The median target sample size was 60 [27,28,29,30,31] (median (min–max)) patients. Participants were adults with American Society of Anesthesiologists physical statuses categorized as classes I to III. The studies investigated patients who underwent surgery in different specialties: abdominal surgery, neurosurgery, orthopedic surgery, ENT surgery, thoracic surgery, and mixed surgeries. The majority of studies were conducted in Asia and Europe. General anesthesia was maintained with halogenated anesthetic agents (*N* = 25) or with an infusion of propofol (*N* = 8). The majority of RCTs (*N* = 27) investigated OFA with an opioid substitute, while seven RCTs did not use an opioid substitute. Five studies allowed for opioid use in the OFA groups only for induction. Twenty-two RCTs explored the use of dexmedetomidine, two RCTs explored combined use with lidocaine, and two RCTs used only ketamine, while other substitutes or combinations were rare. The comparators in most studies were remifentanil (*N* = 19.58%), followed by fentanyl (*N* = 13, 39%) (Table 1). A detailed description of each trial is provided in Supplementary Table S1.

### 3.3. Risk of Bias Assessment of the Included Studies

**Figure 2:**
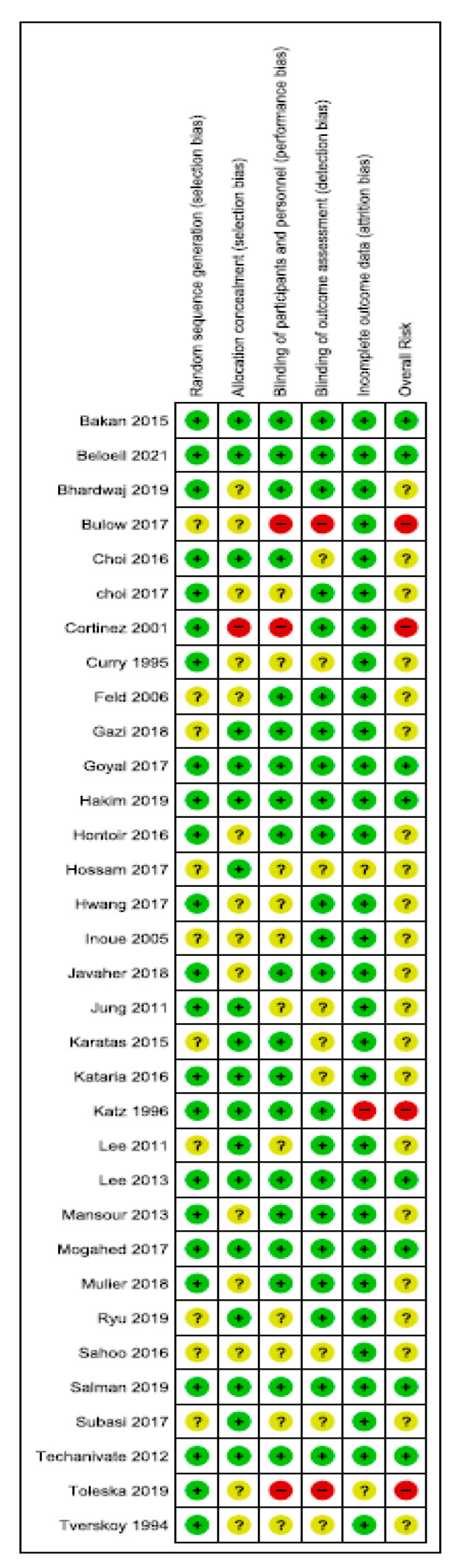
Risk-of-bias summary: review of authors’ judgements about each risk-of-bias item for each included study.

Overall, 8 trials were classified as being at low risk of bias, 21 trials were classified as having unclear bias, and 4 trials were classified as being at high risk of bias. The randomization procedure was adequately described in 22 trials (67%), and concealment of treatment allocation was described in 18 trials (55%). In total, 10 studies (12.5%) were double-blinded, while all others were classified as unclear. Three studies had an unclear or high risk of incomplete data outcomes (Figure 2).

### 3.4. Pain Intensity

Postoperative pain intensity at rest was assessed in 26 trials including 1568 patients compared at 2 h, in 11 trials including 487 patients at 24 h, and in 5 trials including 158 patients at 48 h. Only one trial reported chronic pain. At 2 h, the OFA groups reported significantly lower pain scores at rest than the OBA groups (0.75 (−1.18; −0.32)), but did not reach MCID. The subgroup analyses showed that this difference was based only in the OFA group with an opioid substitute (Supplementary Table S2). There was no significant difference in the decrease in pain between the OFA and OBA groups at 24 h and 48 h. For pain, the evidence qualities were low to moderate (Table 2).

### 3.5. Postoperative Morphine Use

Overall, 13 RCTs including 551 patients reported data on morphine titration in PACU and 10 RCTs including 427 patients reported data on 24 h cumulative morphine use. Less morphine was required by patients in the OFA groups than by patients in the OBA groups at 2 h. However, the difference was very small (1.61 mg (−2.69; −0.53)), with an even greater sparing effect when considering only the active-drug regimen (−2.57 mg, (−3.87; −1.27)). At 24 h, the opioid sparing effect was still statistically significant (−1.73 mg, *p* < 0.05) but did not reach MCID. At 48 h, the sparing effect persisted when considering only the active drug subgroup (−7.54 mg, *p* = 0.04). For all times, the decrease in morphine consumption reached MCID. For morphine consumption, the quality of evidence was low to moderate (Table 2).

### 3.6. Opioid-Related Adverse Events

The number of patients who experienced nausea, vomiting, sedation, or respiratory depression in the postoperative period was reported in 20, 12, 5, and 5 trials, respectively (Table 2).

**Figure 3:**
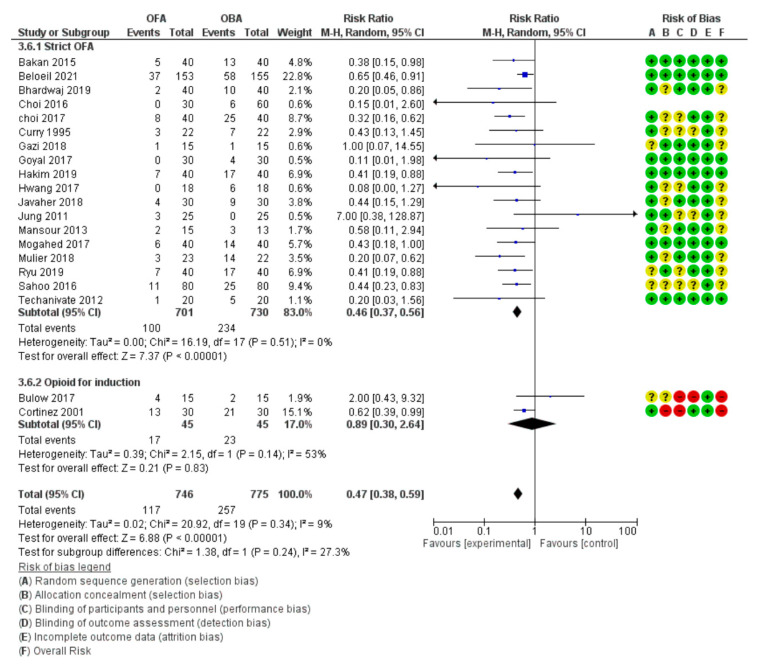
Forest plot for nausea and vomiting in the PACU.

Nausea and vomiting were reported in 20 and 12 studies, respectively. Pooled analysis revealed a significant and important reduction in nausea and vomiting in the OFA group in the PACU compared to in the OBA group, with an RR of 0.46 (0.38, 0.56) and an RR of 0.34 (0.21, 0.56), respectively (Figure 3), which represent relative risk reductions of 54% for nausea and 66% for vomiting. Moreover, this effect persisted in the same proportion at 24 h after surgery, with an RR of 0.55 (0.32, 0.95) for nausea. No differences between subgroups were identified in the subgroup analyses by type of anesthesia, the use of active substitute drugs, or PCA (Supplementary Table S2). However, in the subgroup with the use of opioid during induction, no reductions in nausea and vomiting were reported in the OFA group. For nausea and vomiting, the evidence qualities were moderate to high.

Sedation was reported in five studies. Less sedation was observed by patients in the OFA groups than in the OBA groups in PACU, with an SMD of −0.81 (−1.05, −0.58).

No significant differences were found between the OFA and OBA groups for respiratory depression.

### 3.7. Adverse Effects Linked to Substitution Drug

Less shivering was observed by patients in the OFA groups than in OBA groups in PACU, with an RR of 0.48 (0.33, 0.70). Pooled analysis revealed no significant differences between the OBA group and the OFA group when assessing the incidences of intraoperative tachycardia, bradycardia, hypertension, and hypotension. No difference was reported either when assessing the incidences of postoperative bradycardia and hypotension. For hemodynamic effects, the quality of evidence was very low to moderate (Table 2).

### 3.8. Severe Adverse Events

Only three studies including a total of 417 patients reported the outcome “severe adverse event”. No difference was reported between the OFA and OBA groups. Eight studies including 691 patients reported hemodynamic variation requiring drug administration. No difference was reported between the OFA and OBA groups. For severe adverse events, the quality of evidence was low to moderate (Table 2).

## 4. Discussion

Our meta-analysis found no clinically significant effect of OFA on acute pain and opioid use after surgery in a large sample of studies. However, OFA has a beneficial impact on postoperative comfort with clinically important reductions in postoperative nausea, vomiting, shivering, and sedation incidence. Questions about the potential side effects related to drugs used as a substitute for opioid in OFA persist.

### 4.1. No Clinical Benefit of Opioid-Free Anesthesia on Pain and Morphine Use after Surgery

Our meta-analysis suggests no clinically significant benefit on pain and opioid use after surgery. In fact, the pain intensity reduction is not clinically significant at 2 h (i.e., <10 on a VAS from 0 to 100) and not significantly reduced at 24 h and 48 h after surgery in the OFA group. It therefore does not reach the level of clinically significant reduction defined in our study and validated in the literature [16]. Similarly, the mean reduction in morphine equivalent use does not reach clinical significance at 1, 24, and 48 h after surgery (i.e., 1.6, 1.7, and 7.5 mg, respectively). This morphine sparing effect is well below the reduction of 10 mg for 24 h commonly considered clinically useful to impact the incidence of opioid-related side effects such as PONV and sedation [53,54]. This limited impact of OFA on both pain intensity and morphine use does not support the concept of opioid-induced hyperalgesia, which is an important argument supposedly supporting the OFA rationale [3]. In line with our results, a previous meta-analysis comparing high to low doses of intraoperative opioid impact on postoperative morphine use and pain suggested a non-clinically significant increase in postoperative pain intensity in the high-dose group at 1 h and 24 h after surgery [55]. These combined results support the idea that OFA offers no clinically significant improvement in pain control. Furthermore, it is important to note that the prevention of chronic pain is only explored in one small trial among the 33 included, and that no reduction in the incidence of chronic pain was reported.

### 4.2. OFA Is Associated with an Important Reduction in the Incidence of PONV and a Reduction in Sedation and Shivering

Interestingly, our meta-analysis revealed an intense and prolonged antiemetic effect of OFA compared to opioid-based anesthesia. This effect is clinically important, with a 54% reduction in nausea and a 66% reduction in vomiting in the PACU. This effect compares quite favorably with antiemetic reference treatments such as dexamethasone, droperidol, or ondansetron, which offer 26% reductions in PONV [56]. This effect can be further suggested by the NNTs of 3 and 9 for nausea and vomiting, respectively, in the PACU compared to the estimated NNT of 7 for droperidol and of 5 for ondansetron on vomiting, and the NNT of 5 for droperidol on nausea [57]. These results consider OFA to be quite an effective technique in preventing PONV. Moreover, OFA offers a persistent 45% reduction in nausea at 24 h after surgery, with an NNT of 2. We consider that the level of evidence is moderate to high. The mechanisms potentially involved can be further analyzed with our subgroup analysis. Firstly, it is not related to the postoperative morphine sparing effect that we already described as very low. Indeed, the opioid sparing benefit on PONV incidence has already been largely investigated for the postoperative period. In our meta-analysis, a minimal value of 10 mg of postoperative morphine reduction over 24 h suggests 10% and 3% reductions in nausea and vomiting, respectively [53]. In our review, the morphine sparing effect was much lower (i.e., 1.7 mg at 24 h) and cannot explain the reduction in PONV. Therefore, the reduction in PONV is primarily related to the absence of opioid use during surgery in the OFA group. Secondly, when comparing studies using opioids only for induction with other drugs, the beneficial impact disappears, underlining the apparent necessity to completely avoid intraoperative opioid use to obtain this benefit in PONV reduction. Thirdly, the impact of OFA on NVPO was independent from the type of anesthesia (i.e., halogen vs. propofol), type of postoperative analgesia (i.e., PCA vs. no PCA), and type of drug used for OFA (i.e., dexmedetomidine vs. other drugs, or dexmedetomidine alone or in combination). In conclusion, OFA clearly offers a drastic and prolonged reduction in PONV. This effect is directly related to complete avoidance of opioid use intraoperatively and not to any significant postoperative morphine sparing effect or the impact of substitution drugs.

### 4.3. Other Evidences on the Potential Benefits Related to OFA

We analyzed other postoperative outcomes such as sedation, ileus, respiratory depression, confusion, delirium, shivering, chronic pain after surgery, and opioid misuse. An important reduction in the incidence of sedation in the PACU was observed for patients in OFA groups compared to OBA groups (SMD > 0.7), and a 52% reduction in postoperative shivering was present in the OFA group. The existing literature does not allow for any reliable analysis of the impact of OFA on respiratory depression, confusion, delirium, chronic pain after surgery, and opioid misuse.

### 4.4. OFA May Be Responsible for Additional Side Effects

Does OFA produce new side effects? The POFA trial provides crucial results to this question and offers a more reliable evaluation of the benefit–risk ratio of new strategies in anesthesia management, which is why this large multi-centric study is so precious among OFA studies [31]. In fact, under-evaluation and under-reporting of severe adverse events in the literature about new medications or strategies for OFA are clear limitations to the development of a complete understanding of the pharmacology and related side effects of OFA. In the POFA trial, which is the largest available trial conducted on OFA, the high incidence of bradycardia led to the recommendation of a maximal dose of continuous dexmedetomidine use of 1 mcg/kg/h. Then, after severe adverse events were reported, with five cases of asystolia, patient recruitment was interrupted [31]. The emergence of severe adverse events in a significant cohort underlines that a collection of small studies is unable to offer a reliable evaluation of tolerance issues. In a recent meta-analysis focused on the tolerance of the alpha-2 agonist, hemodynamic adverse effects were observed frequently with the use of dexmedetomidine, especially in cases of bolus or with high-dose continuous infusion, and can be prolonged after discontinuation of it administration, thus requiring monitoring [8]. We highlight that the presence of dexmedetomidine boluses, which were deliberately not used in the POFA study, may have exposed patients to even more severe adverse events. In a subgroup analysis, our quantitative meta-analysis of intra- and postoperative mean arterial blood pressure and heartbeat did not show any differences related to the OFA protocol. However, this result offers weak evidence due to the heterogeneity of OFA opioid substitution protocols and small size trials. In conclusion, uncertainty persists with regard to hemodynamic side effects such as bradycardia for substitution drugs used in OFA. Further studies collecting data on the side effects, especially on severe adverse events, are required to clearly evaluate the benefit–risk ratio for OFA.

### 4.5. Strengths and Limitation of Our Meta-Analysis

Our meta-analysis included a larger sample of studies and patients, with 10 more studies and 905 more patients than the last systematic review published. Our methodology adhered to existing guidelines for quantitative analysis, and we selected only randomized clinical trials clearly investigating OFA versus opioid-based anesthesia. The subgroups allowed us to separately evaluate strict OFA and OFA with opioid use only during induction. Our analysis based on clinical significance offers data that are useful for daily practice. The subgroup analysis supports an in-depth analysis of the antiemetic effect of OFA and provides a better understanding of the mechanisms involved.

We acknowledge the following limitations to our work: Our meta-analysis was based on numerous small trials from single centers. We observed heterogeneity in the protocols used to replace intraoperative opioids and heterogeneity in the protocols used to prevent or treat PONV, and in the tools and timing used for the evaluation of different patient’s outcomes, such as sedation and significant imbalance in terms of the quantity of evidence for each subgroup analyzed. The power and reliability of the pooled estimates could be affected. We observed a substantial between-trial heterogeneity, particularly for the morphine consumption outcome, which was reported previously in other MAs. There is growing evidence that morphine consumption depends more on individual pain vulnerability than on surgical trauma. Indeed, several factors are considered to explain pain vulnerability such as genetics, previous morphine consumption, preoperative chronic pain, and psychological factors. None of these factors were monitored and evaluated in our dataset. Finally, our work did not assess the impact of postoperative multimodal analgesia used in combination, such as nonsteroidal anti-inflammatory drugs.

### 4.6. Should We Use OFA?

According to the results of our meta-analysis, OFA should not be used if the primary goal is to reduce pain and opioid use after surgery [58,59]. This absence of an effect on pain after surgery challenges the clinical significance of opioid-induced hyperalgesia in the acute phase after surgery [60]. In addition, the speculative beneficial impact on the development of chronic pain after surgery could not be confirmed in our review of the existing literature. OFA appears to be useful in reducing postoperative sedation and shivering. However, the most important result is an important and sustained benefit observed as a reduction in PONV when opioid use is completely avoided intraoperatively. This suggests that POFA could be recommended in patients with a high risk of PONV, which may represent one rational suggestion for the use of OFA. Importantly, converging data from the POFA trial and one previous meta-analysis [8,9], although not confirmed by our present meta-analysis, suggest potential severe hemodynamic side effects related to dexmedetomidine. These potential side effects strongly suggest that OFA should not be extensively used before additional data are collected to clarify this issue [59]. What then is the research agenda left for OFA? One can argue that other regimens using less important doses of dexmedetomidine and/or regimens that abstain from a combination with lidocaine may offer more positive benefit–risk ratios. A prolongation of the use of drugs, such as lidocaine, dexmedetomidine, or ketamine, beyond the intraoperative period may also change their impacts on pain, but risk exposure to additional side effects. At this stage, we can only speculate on this matter; therefore, additional trials investigating OFA efficacy and its related side effects are needed.

## 5. Conclusions

In this new meta-analysis exploring the benefits and adverse effects related to opioid-free anesthesia compared to the use of opioid-based anesthesia, no clinically significant benefits were observed in terms of pain and opioid use after surgery. A clear benefit with respect to a reduction in PONV was demonstrated when intraoperative opioids were avoided. Side effects related to dexmedetomidine need to be anticipated, although existing literature does not provide definitive evidence-based conclusions. We conclude that more data on the safe use of OFAs should be collected and that caution is necessary in the development of OFA.

## Figures and Tables

**Table 1:** Characteristics of the 33 trials included in the meta-analysis.

Publication year, median (range).	2015 (1994–2021)
Single-center trials, *n* (%)	32 (96)
Trial size, median (range)	60 (27–316)
Continent of origin	
Europe, *n* (%)	9 (27)
Asia, *n* (%)	15 (45)
North America, *n* (%)	3 (9)
South America, *n* (%)	2 (6)
Africa, *n* (%)	4 (12)
Type of surgery	
Abdominal, *n* (%)	9 (27)
Uro-gynecological, *n* (%)	13 (39)
Orthopedic, *n* (%)	1 (3)
Neurological, *n* (%)	3 (9)
ENT, *n* (%)	4 (12)
Thoracic, *n* (%)	2 (6)
Mixed, *n* (%)	1 (3)
Anesthesia maintenance	
Propofol, *n* (%)	9 (27%)
Halogenated agent, *n* (%)	25 (76%)
OFA group	
Active drug, *n* (%)	24 (72)
Dexmedetomidine and Lidocaine combined	22 (66) 4
Ketamine, *n* (%)	2
Magnesium, *n* (%)	1
Combination, *n* (%)	3 *
Clonidine, *n* (%)	1
Saline, *n* (%)	7 (21)

* Association between ketamine, lidocaine, and magnesium for one trial and dexmedetomidine, ketamine, and lidocaine for two trials.

**Table 2:** Summary of the findings from pairwise meta-analyses and the level of evidence.

Outcomes	Studies	Patients	Effect Size (95% CI) or	Heterogeneity, I^2^ (%)	NNTH (95% CI)	GRADE
*Morphine consumption*						
PACU	13	551	WMD −1.61 (−2.69, −0.53)	86%		⊗⊗⊗ ^Z^
24 h	10	427	WMD −1.73 (−2.82, −0.65)	73%		⊗⊗⊗ ^Z^
48 h	5	318	WMD −3.14 (−10.34, 4.05)	88%		⊗⊗⊗ ^Z^
*Pain score*						
PACU	26	1568	−0.75 (−1.18, −0.32)	92%		⊗⊗⊗ ^Z^
24 h	11	487	0.11 (−0.38, 0.60)	91%		⊗⊗⊗ ^Z^
48 h	5	158	0.16 (−0.09, 0.41)	53%		⊗⊗
*Intraoperative Incidence*						
Tachycardia	2	125	RR 0.89 (0.51; 1.54)	0%	----	⊗ *^Y^
Bradycardia	8	745	RR 0.86 (0.39; 1.92)	55%	----	⊗⊗ ^Z^
Hypertension	4	505	RR 1.07 (0.59; 1.93)	56%	----	⊗⊗ ^YZ^
Hypotension	10	845	RR 0.92 (0.79; 1.08)	46%	----	⊗⊗⊗
*Postoperative Incidence*						
Tachycardia	0	0	Not estimable		----	----
Bradycardia	5	310	RR 1.65 (0.67; 4.08)	0%	----	⊗⊗ *
Hypertension	0	0	Not estimable		----	----
Hypotension	3	200	RR 1.11 (0.21; 5.76)	45%	----	⊗⊗⊗ *^Y^
Nausea, PACU	20	1521	RR 0.46 (0.38; 0.56)	9%	3 (3; 4)	⊗⊗⊗⊗
Nausea, H24	6	450	RR 0.55 (0.32; 0.95)	71%	2 (2; 4)	⊗⊗⊗ ^Z^
Vomiting, PACU	12	788	RR 0.34 (0.21; 0.56)	16%	9 (8; 13)	⊗⊗⊗⊗
Shivering	9	581	RR 0.48 (0.33; 0.70)	12%	6 (5; 10)	⊗⊗⊗
Respiratory depression	5	557	RR 0.51 (0.07; 3.63)	87%	----	⊗⊗ ^Z^*
Cognitive dysfunction	1	308	RR 5.06 (0.25; 105)	---	----	----
Sedation	6	336	SMD −0.81 (−1.05, −0.58)	8%		
Serious adverse events						
Requiring drug administration	8	691	RR 1.23 (0.71; 2.15)	49%	----	⊗⊗⊗
Reported as SAE	3	417	RR 1.47 (0.87; 2.48)	84%	----	⊗⊗⊗ *^YZ^

CI, confidence interval; GRADE, Grading of Recommendations Assessment, Development and Evaluation; NNTH, number needed to treat for an additional harmful outcome calculated only for statistically significant results; RR, risk ratio. The level of evidence was assessed by the GRADE method. ⊗⊗⊗⊗ (High quality): we are very confident that the true effect is close to the estimated effect. ⊗⊗⊗ (Moderate quality): we are moderately confident in the effect estimate; the true effect is likely to be close to the estimated effect, but there is a possibility that it is substantially different. ⊗⊗ (Low quality): our confidence in the effect estimate is limited; the true effect may be substantially different from the estimated effect. ⊗ (Very low quality): we have very little confidence in the effect estimate; the true effect is likely to be substantially different from the estimated effect. * Downgraded for imprecision: optimal information size not reached. ^Y^ Downgraded for insufficient data quality. ^Z^ Downgraded for inconsistency (I^2^ > 50%).

## Supplementary Materials

The following are available online at https://www.mdpi.com/article/10.3390/jcm10102069/s1, Table S1: Trial characteristics; Table S2: Subgroup analysis.
