# Supplementary material for: Opioid-Free Anesthesia Benefit–Risk Balance: A Systematic Review and Meta-Analysis of Randomized Controlled Trials
Source: J Clin Med. 2021 May 12;10(10):2069. doi: 10.3390/jcm10102069 (PMC8150912; doi:10.3390/jcm10102069)
Supplement: Supplementary file 1 [file jcm-10-02069-s001.zip › jcm-1186503-supplementary.pdf]

Supplementary data 1 - Trial characteristics

Reference	Group (n)	Surgery	Opioid regimen<br>Control	 Opioid	Anaesthetic maintenance	PONV prevention	Postoperative analgesia	Outcomes
Bakan et Al. 2015	OFA (43)<br>OBA (42)	Laparoscopic cholecystectomy	Dexmedetomidine<br>I : 0.6 ug/kg in 10 min<br>M : 0.3 ug/kg/h<br><br>Lidocaine<br>I : 1.5 mg/kg<br>M : 2 mg/kg/h	Fentanyl<br>I : 2 ug/kg<br>M : 15 ug/kg/h	Propofol<br>I : 1.5mg/kg<br>M : 10mg/kg/h	8mg i.v Dexamethasone	Fentanyl i.v PCA	Opioid consumption, postoperative pain, side effect
Beloeil et Al. 2021	OFA (158)<br>OBA (158)	Major and intermediate non cardiac surgery	Dexmedetomidine<br>I/M : 0.4 to 1.4 µg/kg/h	Remifentanil<br>I/M : TCI 3 to 5 ng/ml<br><br>i.v Morphine bolus of 0.05 mg/kg at the end of surgery	Desflurane<br><br>Lidocaïne : 1.5 mg/kg/h<br><br>Ketamine : 0.25 mg/kg/h	8mg i.v Dexamethasone	Morphine i.v PCA<br><br>Lidocaïne 1.5 mg/kg/h for 12 hours<br><br>paracetamol 1g/6h<br><br>nefopam 20mg/6h	Opioid side event
Bhardwaj et Al. 2019	OFA (40)<br>OBA (40)	Laparoscopic urological surgery	Dexmedetomidine<br>I : 0.5 µg/kg over 10 min<br>M : 0.1 − 0.3 µg/kg/h<br><br>Lidocaïne<br>I : 1.5 mg/kg<br>M : 0.1 mg/kg/h	Fentanyl<br>I : 2 µg/kg<br>M : intermittent 0.5 µg/kg bolus	Propofol 50−200 µg/kg/min	8mg i.v Dexamethasone<br><br>4mg i.v Ondansetron	75mg i.v diclofenac 1mg i.v. paracetamol<br><br>Rescue : 100mg i.v Tramadol (additional 50 mg every 30 minutes, max 250mg	Opioid consumption, haemodynamic stability
Bulow et Al. 2007	OFA (15)<br>OBA (15)	Gynaecologic videolaparoscopic surgery	Single 100µg of i.v fentanyl at the induction<br><br>Dexmedetomidine<br>M : 0.5µg/kg/h	Remifentanil<br>I/M : 0.3µg/kg/min	Propofol<br>TCI : 4.0-5.0µg/ml	None	100mg i.v ketoprofen<br><br>Rescue 1g i.v Dypirone	Haemodynamic stability, cortisol/glucose level
Choi J.W. et Al. 2016	OFA (32)<br>OBA 1 (31)<br>OBA 2 (30)	Laparoscopic hysterectomy	Dexmedetomidine<br>I : 1 µg/kg over 10 min<br>M : 0.5 µg/kg/h	OBA 1 : Fentanyl<br>I : 1.0 µg/kg<br>M : 0.4 µg/kg/h<br><br>OBA 2 : Remifentanil<br>I : 1.0 µg/kg<br>M : 0.08 µg/kg/min	Desflurane 6-7% volume	None	30mg i.v ketorolac	Haemodynamic stability, sedation, postoperative pain
Choi E.K. et Al. 2017	OFA (40)<br>OBA (40)	Thyroïdectomy	Dexmedetomidine<br>I : 1 µg/kg over 10 min<br>M : 0.3–0.5 µg/kg/h	Remifentanil<br>I : TCI 4 ng/ml<br>M : TCI of 2-3ng/mL	1.0–2.5% Sevoflurane	None	None	PONV
Cortinez et Al. 2001	OFA (30)<br>OBA (30)	Open gynaecological surgery	Sevoflurane<br>I : 2%<br>M : 0.5±1.0% increments	Remifentanil<br>I : 0.25mg/kg/min<br>M : 0.05±0.1mg/kg/min increment/decrement	Sevoflurane	0.2mg i.v Droperidol with each PCA bolus	Morphine i.v PCA (bolus dose 1mg, 8min lockout)	Opioid consumption, postoperative pain

Curry et Al. 1995	OFA (22)<br>OBA (22)	laparoscopic Electrocautery tubal ligation	Saline<br>I : 1 µg /kg	Fentanyl<br>I : 1 µg /kg	Propofol 200-300 µg/kg/min<br><br>Isoflurane not exceeding 0.5%.	None	Fentanyl i.v PCA (25 µg bolus dose, 5 min lockout, no dose limit)	Opioid consumption, postoperative pain
Feld et Al. 2006	OFA (10)<br>OBA (10)	Open gastric bypass	Dexmedetomidine<br>I : 0.5µg/kg over 10min<br>M : 0.4µg/kg/h	Fentanyl<br>I : 0.5µg/kg<br>M : 0.5µg/kg/h	Desflurane	None	Morphine i.v PCA	Heamodynamic stability, postoperative pain
Gazi et Al. 2018	OFA (15)<br>OBA (15)	Hysteroscopy	Dexmedetomidine<br>I/M : 0.2-0.7 µg/kg/h	Remifentanil<br>I/M : 0.05-0.5µg/kg/min	2% Fi sevoflurane	0.1 mg/kg i.v Ondansetron	1000 mg i.v Paracetamol	aIntraoperative pain, haemodynamic stability, postoperative pain, side effect
Goyal et Al. 2017	OFA (30)<br>OBA (30)	Breast cancer	Dexmedetomidine<br>I : 1 µg/kg over 10 min<br>M : 0.25 µg/kg/h	Fentanyl<br>I : 2 µg/kg over 10 min<br>M : 0.5 µg/kg/h	Desflurane	None	1mg/kg i.v Diclofenac and 1g i.v Paracetamol<br><br>Rescue :<br>1mg/kg i.v tramadol and 10mg i.v Pethidine (max 50 mg) as second rescue analgesia	Opioid consumption, haemodynamic stability, halogene requirement, recovery
Hakim et Al. 2019	OBA (40)<br>OFA (40)	Gynecological laparoscopic surgery	Dexmedetomidine<br>I : 0.6 µg/kg over 5 min<br>M : 0.2µg/kg/h ±0.1µg/kg/h	Fentanyl<br>I : 1 µg/kg over 5 min<br>M : 0.5µg/kg/h ±0.1µg/kg/h	Propofol<br>I/M : 5–10 mg/kg/h	None	30mg i.v Ketorolac, and 1g i.v acetaminophen every 8 h<br><br>Rescue : 0.5 µg/kg i.v Tramadol	Recovery
Hontoir et Al. 2016	OFA (33)<br>OBA (33)	Breast cancer surgery	Clonidine<br>I : 0.2 µg/kg	Remifentanil<br>I/M : TCI	Sevoflurane	4mg i.v Ondansetron	1g i.v Paracetamol and 75mg i.v Diclofenac and 0.03mg/kg i.v Piritramide<br><br>Piritramide i.v PCA (2 mg bolus, 10min lockout interval, maximum dose of 20mg/4h)	Recovery, opioid consumption, postoperative pain
Hossam et Al. 2017	OFA (30)<br>OBA (30)	Arthroscopic shoulder surgery	Dexmedetomidine<br>I : 1µg/kg over 10min<br>M : 0.5µg/kg/h	Fentanyl<br>I : 1µg/kg<br>M : 0.5µg/kg/h	1.5% isoflurane	None	Rescue : 0.04 mg/kg i.v morphine bolus	Haemodynamic stability, postoperative pain, sedation
Hwang et Al. 2017	OFA (20)<br>OBA (20)	Posterior lumbar interbody fusion	Dexmedetomidine<br>I/M : 0.01-0.02µg/kg/min	Remifentanil<br>I/M : 0.01-0.02 µg/ kg/min	propofol<br>I/M : 3-12 mg/kg/h	0.3mg i.v Ramosetron	i.v Hydromorphone PCA (background rate of 0.12mg/h, 0.12mg bolus	Opioid consumption,

							dose, 10min lockout interval of 10 minutes<br><br>Rescue : 1 µg/kg i.v Fentanyl or 50 mg i.v Tramadol	postoperative pain, PONV
Inoue et Al. 2005	OFA (25)<br>OBA (25)	Cervical spine surgery	Single 100µg i.v fentanyl bolus at the induction<br><br>M : 1.5%-2.5% Fet Sevoflurane	Fentanyl<br>I : 100µg i.v<br>M : 50µg intermittent bolus + 0.5-1.0% Fet Sevoflurane	Sevoflurane	None	50mg i.v Flurbiprofen axetil<br><br>Rescue : 50µg i.m Fentanyl	Recovery
Javaherforoo-shzadeh et Al. 2018	OFA (30)<br>OBA (30)	Lumbar discopathy surgery	Fentanyl<br>I : 2 to 3µg/kg<br><br>Dexmedetomidine<br>M : 0.3 to 0.7µg/kg/h	Fentanyl<br>I : 2 to 3µg/kg<br><br>Remifentanil<br>M : 0.1 to 1.0µg/kg/h	Propofol 50 to 100µg/kg/min	None	None	Haemodynamic stability, blood loss
Jung et Al. 2011	OFA (25)<br>OBA (25)	Elective total laparoscopic hysterectomy	Dexmedetomidine<br>I : 1 µg/kg over 10 min<br>M : 0.2-0.7 µg/kg/h	Remifentanil<br>I : 0.8-1.2 µg/kg over 1 min<br>M : 0.05-0.1 µg/kg/h	6-7 vol.% of desflurane	None	30mg i.v ketorolac	heamodynamic stability, sedation, postoperative pain
Kataria et Al. 2016	OFA (30)<br>OBA (30)	Laparoscopic cholecystectomy	Dexmedetomidine<br>I : 1 µg/kg<br>M : 0.2 µg/kg	Fentanyl<br>I : 1 µg/kg<br>M : 0.2 µg/kg intermittent bolus.	Isoflurane	None		haemodynamic stability
Katz et Al. 1996	OFA (15)<br>OBA (15)	Hysterectomy	Saline	Alfentanil<br>I : 30ug/kg<br>M : 10-20ug/kg every hour	Isoflurane	None	Morphine i.v PCA (1,5-2.0mg bolus, 5-7min lockout intervalle, maximum dose of 30 mg/4h)	Opioid consumption, postoperative pain
Lee C. et Al. 2011	OFA (30)<br>OBA (30)	Tonsillectomy	Saline	Remifentanil<br>I : 1 µg/kg<br>M : 0.1 µg/kg/min ± 0.05 µg/kg/min increment	Sevoflurane<br>OFA titrated on BIS<br>OBA 1 MAC	None	Rescue : 25mg i.v Pethidine 25 mg (VAS ≥ 4) or 30mg i.v Ketorolac (VAS ＜ 4)	Opioid consumption, postoperative pain
Lee J. et Al. 2013	OFA (32)<br>OBA (34)	Endoscopic sinus surgery	Dexmedetomidine<br>I : 1 µg/kg over 10min<br>M : 0.4-0.8 µg/kg/h.	Remifentanil<br>I : 1 µg/kg over 1 min<br>M : 0.2-0.4 µg/kg/min	5 to 7 vol% Desflurane	None	Rescue : 30mg i.v ketorolac tromethamine 30 mg	Haemodynamic stability
Mansour et Al. 2013	OFA (15)<br>OBA (13)	Laparoscopic sleeve surgery	Ketamine<br>I : 0.5 mg/kg<br>M : 0.5 mg/kg/h (IBW)	Fentanyl<br>I : 2-5 µg/kg<br>M : 0.025-0.25µg /kg/min (IBW)	2-4% sevoflurane	50mg i.v Ranitidine + 10mg i.v Metoclopramide + 8mg i.v Dexamethasone	OFA : 1g/6h i.v Paracetamol and 75mg/12h i.m and 60-100mg/12h i.v and Tramadol i.v PCA (10mg bolus, 6min lock-out interval, no basal infusion).	Haemodynamic stability

							Rescue : 2-4m/2h i.v Morphine<br><br>OBA : 1g/6h i.v Paracetamol and Fentanyl i.v PCA (10µg bolus, 6min lock-out interval)	
Mogahead 2017	OFA (40)<br>OBA (40)	Laparoscopic cholecystectomy	Dexmedetomidine<br>I : 0.7 ug/kg over 10 min M : 0.4 ug/kg/h	Remifentanil<br>I : 0.7 ug/kg over 10 min<br>M : 0.2 ug/kg/h	1-1.5MAC Sevoflurane	8mg i.v Dexamethasone and 4mg i.v Ondansetron	Rescue : 25 mg i.v fentanyl	Expiratory fraction Sevoflurane concentration
Mulier et Al. 2018	OFA (25)<br>OBA (25)	laparoscopic bariatric surgery	Dexmedetomidine<br>I : 0.5 mg/kg over 10 min<br>M : 0.25 - 1 mg/<br>kg/h<br><br>Ketamine<br>I : 0.25 mg/kg<br><br>Lidocaine<br>I : 1.5 mg/kg<br>M : 1.5-3 mg/kg/h	Sufentanil<br>I : 0.5 mg/kg<br>M : 0.25-1 mg/kg/ (IBW)	Sevoflurane	None	2g i.v Paracetamol then 1g/6h<br>And Morphine i.v PCA (2mg bolus, 5min lock-out interval, no basal infusion)	Haemodynamic stability, opioid consumption, recovery
Ryu et Al. 2019	OFA (40)<br>OBA (40)	Middle ear surgery	Magnesium sulfate<br>I : 50mg/kg over 10 min<br>M : 15mg/kg/h	Remifentanil<br>I : TCI 4ng/ml<br>M : TCI 3-4 ng/ml	Sevoflurane	None	30mg i.v Ketorolac	Haemodynamic stability, postoperative pain
Sahoo et Al. 2016	OFA (80)<br>OBA (80)	Laparoscopic gynaecological surgery	Dexmedetomidine<br>I : 0.01µg/kg/min<br>M : 0.05 µg/kg/min	Remifentanil<br>I : 0.01 µg/kg/min<br>M : 0.05µg/kg/min	Propofol 3-12 mg/kg/h	0.3mg i.v Ondansetron	Morphine i.v PCA (2 mg bolus, background rate of 1 mg/h, 10min lockout interval)<br>Rescue : 1 µg/kg i.v Fentanyl or 50mg i.v Tramadol	Opioid consumption, postoperative pain, PONV
Salman et Al. 2019	OFA (30)<br>OBA (30)	Ambulatory gynaecologic laparoscopic surgery	Dexmedetomidine<br>I : 1 µg/kg over 10min<br>M : 0.4 µg/kg/h	Remifentanil<br>I : 1 µg/kg over 10 min<br>M : 0.2 µg/kg/min	6% desflurane	None	1g p.o Paracetamol<br><br>Rescue : 30-40 µg /kg morphine	Haemodynamic stability, opioid consumption, postoperative pain, PONV, recovery
Senol Karatas et Al.<br>2015	OFA (16)<br>OBA 1 (16)<br>OBA 2 (16)<br>OBA 3 (16)	major abdominal surgery	Saline<br>I : 5 cc bolus<br>M : 10 cc/h	OBA 1 : Remifentanil<br>I : 1µg/kg<br>M : 0.25 µg/kg/min<br><br>OBA 2 : Alfentanil<br>I : 10 µg/kg	1 MAC desflurane	None	1 g i.v Paracetamol and 1 mg/kg i.v Mepiridine<br><br>Meperidine i.v PCA (15mg bolus dose, 8min lockout time)	Opioid consumption

				M : 0.50 µg/kg/min<br><br>OBA 3 : Morphine<br>I : 0.1 mg/kg<br>M : 0.02 mg/kg/h				
Subasi et Al. 2017	OFA (20)<br>OBA (20)	Laparoscopic cholecystectomy	Single 1µg/kg i.v Fentanyl bolus at the induction<br><br>Dexmedetomidine<br>M : 0.5µg/kg/h lowered to 0.3 µg/kg/h after 5 minute of infusion	Fentanyl<br>I : 1µg/kg<br>M : 0.5µg/kg/h lowered to 0.3 µg/kg/h after 5 minute of infusion	Propofol 150 µg/kg/min	None	Morphine i.v PCA (1mg bolus dose, 20min lockout interval)	Haemodynamic stability, opioid consumption, postoperative pain, recovery
Techanivate et Al. 2012	OFA (20)<br>OBA (20)	Outpatient gynaecologic laparoscopy	Dexmedetomidine<br>I : 0.5 µg/kg<br>no maintenance	Fentanyl<br>I : 0.5 µg/kg<br>no maintenance	desflurane	None	1g p.o paracetamol<br>Rescue : 25µg i.v Fentanyl.	Postoperative pain
Toleska et Al. 2019	OFA (30)<br>OBA (30)	Laparoscopic cholecystectomy	Ketamine<br>I : 0.5 mg/kg<br><br>Lidocaïne<br>M : 2 mg/kg/h<br><br>Magnesium sulphate<br>M : 1.5 g/h	Fentanyl :<br>I : 2µg/kg<br>M : intermittent Fentanyl bolus	0.7-1 MAC Sevoflurane	0.1 mg/kg i.v Dexamethasone	1g i.v Paracetamol, and 2.5g Metamizole (OBA)<br><br>Rescue : 100mg i.v Ketoprofen or 100mg i.v Trodon	Opioid consumption, postoperative pain
Tverskoy et Al. 1994	OFA 1 (9)<br>OFA 2 (9)<br>OBA (9)	Elective transabdominal hysterectomy	OFA 1 : ketamine<br>I : 2 mg/kg<br>M : 20 pg/kg/min<br><br>OFA 2 : isoflurane only	Fentanyl<br>I : 5 pg/kg<br>M : 0.02 pg/kg/min	Isoflurane	None	First 9h : i.v meperidine on demand<br>9 to 24h : 50mg/4h i.m Meperidine ± 25 mg on demand<br>> 24h : 0.5g/4h p.o Dipyrone	Postoperative pain

OFA : Opioïd-free anaesthesia ; OBA : Opioïd based anaesthesia ; PONV : Postoperative nausea and vomiting ; I : induction period ; M : maintenance period ; i.v : intravenous ; i.m : intramuscular ; PCA : patient-controlled analgesia ; TCI : target-controlled infusion

Supplementary data 2 : Subgroup analysis

Subgroup analysis for morphine at 24h

Outcomes	Number of trials	Number of participants	SMD Random effect [95% CI]	Heterogeneity (I^2)	Heterogeneity (I^2) – Test for subgroup differences
Active drug	5	200	-3.84 [-5.03, -2.65]	0%	91%
Saline	5	227	0.84 [-1.70, 3.39]	66%	
Strict OFA	7	327	-1.58 [-3.64, 0.48]	72%	0%
Opioid during induction	2	100	-2.86 [-6.20, 0.48]	33%	
PCA	7	340	-2.65 [-5.20, -0.11]	74%	74%
No PCA	2	87	0.10 [-3.55, 3.76]	66%	
Propofol	2	77	-4.17 [-6.19, -2.15]	19%	74%
halogenated agents	7	350	-0.89 [-2.76, 0.99]	62%	

Subgroup analysis for pain at 24h

Outcomes	Number of trials	Number of participants	SMD<br>Random effect<br>[95% CI]	Heterogeneity (I^2)	Heterogeneity (I^2) – Test for subgroup differences
Active drug	9	547	0.02 [-0.88, 0.91]	94%	0%
Saline	2	100	0.23 [-0.28, 0.74]	74%	
Strict OFA	9	547	0.05 [-0.70, 0.79]	93%	0%
Opioid during induction	2	100	-0.25 [-0.43, -0.06]	0%	
PCA	8	500	-0.46 [-0.98, 0.06]	86%	71%
No PCA	3	147	1.04 [-0.44, 2.53]	95%	
Propofol	3	237	-1.00 [-2.61, 0.61]	91%	48%
Halogenated agents	8	410	0.23 [-0.45, 0.91]	92%	

Subgroup analysis for nausea at PACU

Outcomes	Number of trials	Number of participants	RR Random effect [95% CI]	Heterogeneity (I^2)	Heterogeneity (I^2) – Test for subgroup differences
Active drug	18	1417	0.45 [0.37, 0.55]	13%	0%
Saline	2	104	0.57 [0.36, 0.90]	0%	
Strict OFA	17	1371	0.31 [0.22, 0.43]	12%	15%
Opioid during induction	3	150	0.52 [0.19, 1.47]	23%	
PCA	7	717	0.37 [0.26, 0.52]	12%	71%
No PCA	13	804	0.31 [0.21, 0.46]	15%	
Propofol	7	526	0.32 [0.20, 0.50]	10%	15%
Halogenated agents	13	995	0.35 [0.26, 0.48]	23%	
